# *O*-GlcNAcylation-regulated classical programmed cell death in diseases: molecular crosstalk and therapeutic opportunities

**DOI:** 10.3389/fimmu.2025.1658769

**Published:** 2025-11-06

**Authors:** Runyuan Liu, Jingxuan Wei, Zhengqing Luo, Xinyi Gao, Hongshuo Zhang, Ying Kong

**Affiliations:** 1 Department of Biochemistry and Molecular Biology, College of Basic Medical Sciences, Dalian Medical University, Dalian, China; 2 Advanced Institute for Medical Sciences, Dalian Medical University, Dalian, China

**Keywords:** *O*-GlcNAc, apoptosis, autophagy, pyroptosis, ferroptosis, necroptosis

## Abstract

*O-*linked β-N-acetylglucosamine (*O-*GlcNAc) is a reversible post translational modification (PTM) involving the attachment of β-N-acetylglucosamine to serine or threonine residues of target proteins. This modification regulates a wide range of cellular functions, including signal transduction, gene expression, protein stability, and cellular metabolism. However, the regulatory patterns of *O*-GlcNAc in cell death have not been thoroughly summarized or extensively discussed, and detailed mechanistic studies remain limited. This review provides an updated overview of recent advances linking *O*-GlcNAc with principal types of programmed cell death (PCD), including apoptosis, autophagy, pyroptosis, ferroptosis, and necroptosis. The occurrence of these forms of PCD plays a critical role in exacerbating immune-inflammatory diseases, neurodegenerative disorders, organ and tissue injury, cardiovascular diseases, and metabolic diseases, whereas in cancer, the induction of PCD can inhibit tumor initiation and progression. Therefore, we focus on the emerging roles of *O*-GlcNAc in modulating principal types of PCD in these diseases and discuss its potential as a therapeutic target.

## Graphical Abstract

*O-*GlcNAcylation in the regulation of PCD.

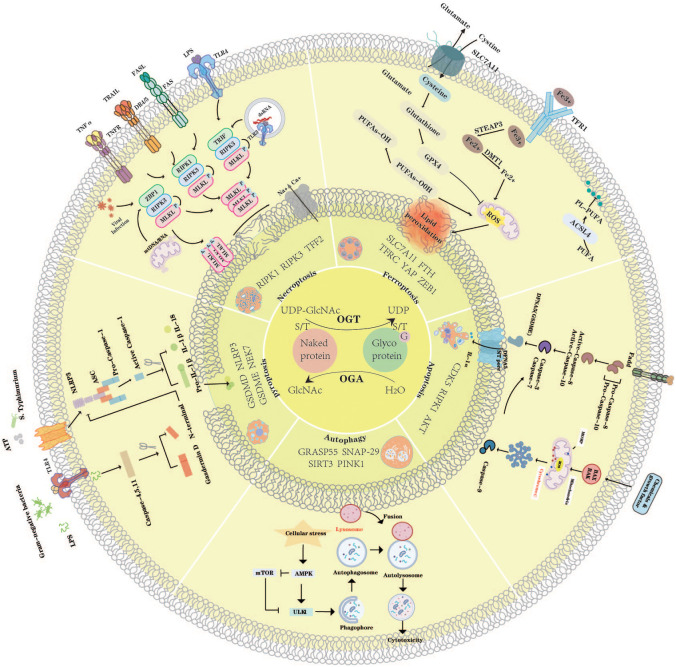

## Introduction

PCD is a tightly regulated biological process that occurs in an orderly manner in all organisms and is essential for maintaining cellular homeostasis (1). With over 50 billion cells undergoing PCD daily in the human body, gaining deeper insight into this process is crucial. This genetically regulated and orderly form of cell death, specifically apoptosis, was initially identified in insects (1965) (2) and later characterized in mammals (1972) (3). PCD is essential for normal development and homeostasis, and is involved in numerous biological and pathological contexts (4). Several distinct forms of PCD, including apoptosis, autophagy, pyroptosis, ferroptosis, and necroptosis (5), play a crucial role in the initiation and progression of immune-inflammatory diseases, neurodegenerative diseases, tissue injury, cardiovascular diseases, cancer, and metabolic diseases. In immune-inflammatory and metabolic diseases, excessive PCD disrupts immune homeostasis and promotes chronic inflammation (6, 7). In neurodegenerative diseases, aberrant apoptosis and autophagy lead to neuronal loss and protein aggregation (8). In cardiovascular diseases and tissue injury, uncontrolled apoptosis, ferroptosis, and pyroptosis exacerbate cellular damage and impair tissue repair (1, 9). Conversely, in cancer, the induction of PCD suppresses tumor initiation and progression (10). Therefore, understanding the mechanisms underlying PCD regulation provides a crucial basis for developing therapeutic strategies across these disease contexts.

*O-*GlcNAcylation not only regulates cell growth, proliferation, metabolism, and function but also plays a crucial role in modulating PCD pathways, thereby influencing the pathogenesis of multiple diseases (11). However, the regulation of PCD by *O-*GlcNAcylation is not a simple binary process. Its effects are highly context-dependent, varying with cell types, disease states, target proteins, and the surrounding microenvironment. Therefore, this complex regulatory mechanism remains to be summarized. This review systematically synthesizes the regulatory networks through which *O*-GlcNAcylation modulates apoptosis, autophagy, ferroptosis, pyroptosis, and necroptosis across different disease contexts, addressing a gap in the literature where these processes have not been comprehensively analyzed together. In this review, we present a novel and comprehensive summary of the dual role of *O*-GlcNAcylation in either promoting or suppressing PCD across diverse pathological contexts, including immune-inflammatory disorders, neurodegenerative diseases, organ and tissue injury, cardiovascular diseases, cancer, and metabolic disorders. Importantly, we highlight the regulatory mechanisms, therapeutic potential, and limitations of *O*-GlcNAcylation-regulated PCD, offering a framework for future research aimed at targeting *O*-GlcNAcylation-regulated PCD in disease treatment.

## *O-*GlcNAcylation

*O-*GlcNAcylation, a dynamic post-translational modification of proteins, was first discovered by Torres and Hart in 1984 during their investigation of glycosylation in lymphocytes (12). *O*-GlcNAcylation predominantly occurs in the nucleus, cytoplasm, and mitochondria, depending on the subcellular localization of its target proteins (13). *O-*GlcNAc acts as a cellular nutrient sensor that is dynamically regulated by *O-*GlcNAc transferase (OGT) and *O-*GlcNAcase (OGA) (14). OGT is responsible for catalyzing *O*-GlcNAc modification of target proteins. In contrast, OGA catalyzes the hydrolytic removal of *O-*GlcNAc, maintaining the dynamic cycling of this modification in response to cellular metabolic states (15). Glucosamine (GlcN) and N-acetylglucosamine (GlcNAc) act as critical intermediates in the hexosamine biosynthetic pathway (HBP), which is regulated by Glucose: Fructose-6-phosphate amidotransferase (GFAT) (16). In this pathway, GFAT mediates the formation of glucosamine-6-phosphate (GlcN-6-P) from fructose-6-phosphate and glutamine, which is then processed to uridine 5′-diphosphate-N-acetylglucosamine (UDP-GlcNAc). OGT uses UDP-GlcNAc to attach a single GlcNAc unit to serine or threonine residues of nuclear and cytoplasmic proteins, thereby regulating *O*-GlcNAc modification and associated cellular responses (17).

*O*-GlcNAcylation closely interacts with other PTMs such as phosphorylation, ubiquitination, and acetylation. For instance, *O*-GlcNAcylation and phosphorylation often occur on the same or adjacent ser/thr residues, and the two modifications can either promote each other or competitively antagonize one another. This crosstalk is particularly prominent on metabolic enzymes controlling glucose homeostasis, fine-tuning protein activity and stability in response to cellular signals (18). *O*-GlcNAcylation and ubiquitination are interdependent: *O*-GlcNAcylation can modulate protein ubiquitination and stability, while ubiquitination can regulate OGT levels, together affecting protein function and cellular processes (19). In addition, elevated *O*-GlcNAcylation can promote protein acetylation, while acetylation in turn may modulate OGT and OGA activity, thereby influencing the dynamic cycling of *O*-GlcNAc (20). Through these competitive and cooperative interactions, *O*-GlcNAcylation integrates multiple cellular signals to precisely regulate protein functions.

### Biological function of O-GlcNAcylation

**Figure 1:**
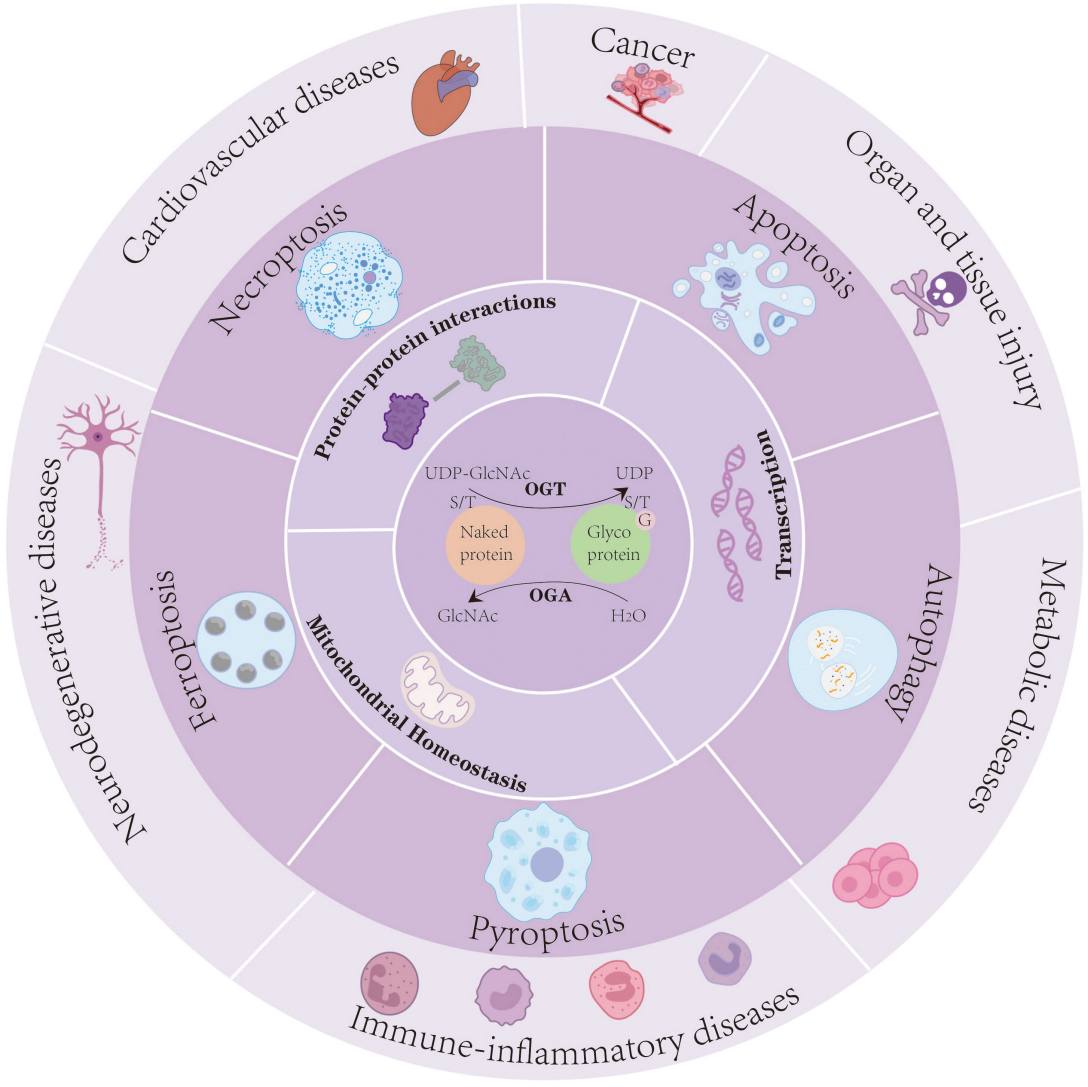
*O*-GlcNAcylation serves as a dynamic nutrient and stress sensor that regulates protein–protein interactions, transcriptional activity, and mitochondrial homeostasis. Through these mechanisms, *O*-GlcNAc modification orchestrates multiple PCD pathways-including apoptosis, autophagy, ferroptosis, pyroptosis, and necroptosis-to modulate cellular fate under pathological conditions. Dysregulated *O*-GlcNAc signaling contributes to the onset and progression of immune-inflammatory diseases, neurodegenerative disorders, cardiovascular diseases, cancers, organ and tissue injury, and metabolic disorders.

*O*-GlcNAc is involved in modulating a wide range of cellular processes by influencing both the molecular properties of target proteins and the downstream cellular pathways they regulate (**Figure 1**) (21).

*O*-GlcNAcylation serves as a dynamic nutrient and stress sensor that regulates protein–protein interactions, transcriptional activity, and mitochondrial homeostasis. Through these mechanisms, *O*-GlcNAc modification orchestrates multiple PCD pathways-including apoptosis, autophagy, ferroptosis, pyroptosis, and necroptosis-to modulate cellular fate under pathological conditions. Dysregulated *O*-GlcNAc signaling contributes to the onset and progression of immune-inflammatory diseases, neurodegenerative disorders, cardiovascular diseases, cancers, organ and tissue injury, and metabolic disorders.

#### Molecular effects of O-GlcNAcylation on target proteins

##### Protein−protein interactions

*O*-GlcNAcylation profoundly influences the expression, stability, and signaling activities of target proteins, thereby modulating their biological functions. This modification often exerts its regulatory effects through crosstalk with other PTMs. Studies have shown that *O*-GlcNAc modification of target proteins can regulate their stability and downstream signaling functions by modulating the crosstalk between *O*-GlcNAcylation and other PTMs, including phosphorylation, ubiquitination, and acetylation. For instance, *O-*GlcNAc modification of CK2α at Ser 347 disrupts its interaction with the downstream protein Pin1 through the proteasomal pathway, leading to reduced phosphorylation, which in turn reduces protein stability (22). Similarly, *O-*GlcNAc of RIPK1 at Ser 331, Ser 440, and Ser669 regulates RIPK1 ubiquitination, attenuates RIPK1/FADD/Caspase-8 complex formation, and consequently promotes NF-κB activation (23). In contrast, *O-*GlcNAc of β-arrestin-1 enhances its stability and interaction with p300, facilitating histone H4K16 acetylation, activating the mTOR pathway, and suppressing podocyte autophagy (24). Moreover, *O-*GlcNAcylation influences the regulation of metabolic pathways and cell death-related processes mediated by target proteins. For example, although elevated transferrin receptor (TFRC) expression increases iron uptake and promotes ferroptosis, *O*-GlcNAc modification at Ser 687 enhances its interaction with the E3 ubiquitin ligase MARCH8, thereby reducing iron transport (25). In conclusion, an in-depth understanding of the effects of *O-*GlcNAc modification on target proteins is essential for elucidating disease mechanisms and advancing the development of targeted therapeutic strategies, including small-molecule inhibitors or modulators of OGT/OGA, interventions targeting specific signaling pathways affected by *O*-GlcNAcylation, and approaches manipulating *O*-GlcNAc at defined protein residues (26, 27).

##### Transcription

Typically, *O-*GlcNAcylation regulates transcription by directly modifying transcription factors, modulating promoter activity, and orchestrating chromatin remodeling. For instance, *O-*GlcNAc modification of the transcription factor STAT6 enhances its transcriptional activity, leading to upregulation of the GSDMC gene family and promoting membrane pore formation, which facilitates IL-33 secretion (28). Similarly, *O-*GlcNAcylation of β-catenin stabilizes the protein by preventing its interaction with β-TrCP, thereby protecting it from proteasomal degradation. This modification also enhances its interaction with EZH2, promotes EZH2 recruitment to promoter regions, and induces substantial changes in the transcriptomic profile (27). Moreover, *O-*GlcNAc of ZNF263 at Ser 662 promotes its chromatin association with OGT and facilitates its binding to the promoters of genes such as DOCK7, NPTX1, and UFSP2, thereby influencing hepatocellular carcinoma (HCC) progression (29).

Additionally, *O-*GlcNAc modification of the chromatin remodeler MORC2 at Thr556 is essential for the TGF-β1-induced transcriptional activation of target genes such as CTGF and SNAIL. Mutation of this residue disrupts MORC2-dependent processes, including breast cancer cell motility, invasion, and pulmonary metastasis (30). Collectively, these findings underscore the critical role of *O*-GlcNAcylation in transcriptional regulation and highlight its potential as a therapeutic target in various diseases.

#### Cellular functions regulated by O-GlcNAcylation

Beyond the direct modification of individual proteins, *O*-GlcNAcylation also exerts broad effects on cellular homeostasis. Numerous studies have summarized its roles in regulating cell proliferation, migration, invasion, and various forms of cell death (31). Rather than reiterating these findings, here we focus on its regulatory functions in mitochondrial dynamics, metabolic reprogramming, and mitophagy.

##### Mitochondrial homeostasis

Accumulating research highlights the critical role of *O-*GlcNAcylation in maintaining mitochondrial homeostasis and function (32). *O-*GlcNAc modifications of mitochondria-associated proteins can modulate mitochondrial reprogramming and cellular functions (33). Mitochondrial reprogramming, in turn, refers to the dynamic process by which cells adapt to various physiological or pathological conditions by remodeling mitochondrial metabolism, dynamics, and signaling functions to respond to environmental changes. Importantly, *O*-GlcNAcylation contributes to this process through multiple mechanisms. For example, the mitochondrial isoform OGT (mOGT) has been reported to control mitochondrial morphology and dynamics through Drp1-dependent fission, regulate membrane potential and oxidative phosphorylation, and thereby preserve mitochondrial structure and cell viability (34). *O-*GlcNAcylation of the mitochondrial fission protein Drp1 modulates mitochondrial distribution, particularly during aging (35). Hexokinase (HK), a key rate-limiting enzyme in glycolysis, is anchored to the mitochondrial membrane. *O*-GlcNAcylation of HK1 promotes the formation of a glycolytic metabolon, strengthens HK1-mitochondria interactions, and coordinates glycolysis with mitochondrial ATP production, thereby influencing neuronal metabolism and mitochondrial function (36). Disruption of *O*-GlcNAcylation has been implicated in mitochondrial dysfunction. In cardiomyocytes, moderate increases in protein *O-*GlcNAc contribute to cardiac adaptation. However, long-term increases in protein *O-*GlcNAc lead to mitochondrial dysfunction, cardiac hypertrophy, fibrosis, and diastolic dysfunction (37).

*O*-GlcNAcylation is also essential for mitophagy. Mitophagy is the process by which the autophagic system identifies and removes damaged mitochondria, directing them to lysosomes for degradation (38). Studies have shown that *O*-GlcNAc can affect mitochondrial homeostasis by modulating mitophagy (39). Specifically, *O-*GlcNAc enhances the mitochondrial levels of the mitophagy protein PTEN-induced kinase 1 (PINK1) and the autophagy-related light chain 3 (LC3) through its interaction with PINK1 and is indispensable for maintaining mitochondrial homeostasis, primarily via the PINK1-mediated mitophagy (40, 41). However, the relationship between *O-*GlcNAc modification and mitophagy remains largely unexplored, with current research still in its early stages. Further investigations are needed to elucidate the precise molecular mechanisms and uncover their broader physiological significance. In addition, studies have shown that *O*-GlcNAc modification plays a critical role in regulating mitochondrial-mediated apoptosis. For example, *O*-GlcNAc-modified Txnip can interact with apoptotic pathways, indirectly modulating the balance of Bax and Bcl-2, thereby influencing mitochondrial apoptosis (42). Similarly, enhanced *O*-GlcNAc signaling via GlcN promotes *O*-GlcNAcylation of Akt and Ser473 phosphorylation, leading to increased Bcl-2, decreased Bax and cleaved caspase-3, and protection of mitochondrial function. Although Bax and Bcl-2 have not been reported as direct *O*-GlcNAc targets, these studies indicate that *O*-GlcNAc can indirectly regulate mitochondrial apoptotic pathways through upstream modulators, highlighting its role in cellular protection (43). Together, these findings highlight the critical role of *O*-GlcNAcylation in regulating mitochondrial quality control and apoptosis, setting the stage for its broader involvement in classical forms of PCD.

## *O*-GlcNAcylation-Modulated Classical Forms of Programmed Cell Death

To maintain physiological processes and tissue homeostasis, certain cells in the body must undergo PCD. These cells are damaged, removed, and replaced by new, healthy cells through tightly regulated mechanisms (44, 45). The delicate equilibrium between cell death, proliferation, and differentiation is crucial for various physiological functions, including organ development and cell renewal. However, PCD typically arises as a response to cellular stress, injury, or infection, and is strongly linked to tissue damage and the progression of diseases. Studies have demonstrated that *O-*GlcNAc not only regulates functions such as transcription, metabolism, cell cycle, and differentiation, but also modulates apoptosis, autophagy, ferroptosis, pyroptosis, and necroptosis.

### Apoptosis

The term “apoptosis” was initially proposed in 1972 to describe an active, programmed form of cell death. Apoptotic cells exhibit characteristic features, including cell shrinkage, pyknosis, karyorrhexis, and apoptotic body generation (3). The extrinsic route, which is mediated by the TNF receptor superfamily, is activated through ligand binding to death receptors (46). The intrinsic pathway, regulated by the BCL-2 family, responds to cellular stress and operates through the mitochondrial pathway (47). As key executors, cysteine-dependent aspartate-directed proteases (caspases) drive apoptosis through selective activation. In humans, seven caspases regulate this process (48). Caspases-2, -8, -9, and -10 serve as initiators, whereas caspases-3, -6, and -7 function as effectors executing cell death (49). Among them, the executioner caspases, caspase-3 and caspase-7, are activated through precise cleavage at internal aspartate residues by the initiator caspase-9, following the formation of the apoptosome complex. This process is tightly regulated and necessitates the formation of a multicomponent apoptosome complex, orchestrated by Apaf-1 and cytochrome c, which is released from mitochondria under apoptotic conditions, in the presence of ATP (50, 51). Once activated, effector caspases execute a broad-spectrum proteolysis of key cellular substrates, driving irreversible commitment to apoptotic cell death (52). Essential for maintaining homeostasis, apoptosis regulates cell turnover, embryonic development, tissue differentiation, and regeneration. It is also implicated in pathological processes such as microenvironmental disruption, DNA damage, and tumor metastasis (53).

**Figure 2:**
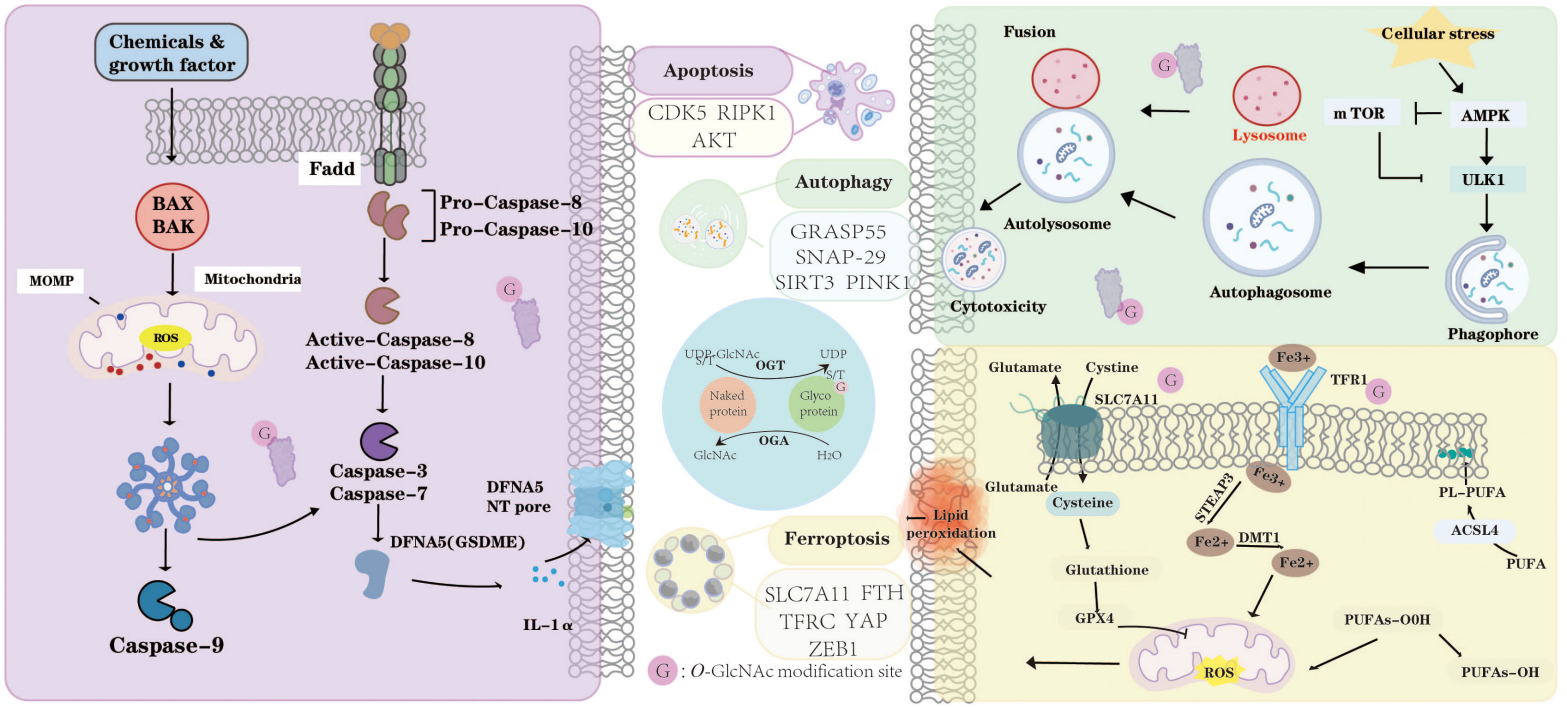
*O*-GlcNAcylation dynamically modulates apoptosis, autophagy, and ferroptosis. In apoptosis, *O*-GlcNAc modification of key proteins such as CDK5, RIPK1, and AKT regulates mitochondrial outer membrane permeabilization (MOMP), caspase activation, and the balance between cell survival and death. In autophagy, *O*-GlcNAcylation of GRASP55, SNAP29, SIRT3, and PINK1 influences autophagosome formation, lysosomal fusion, and mitochondrial quality control under cellular stress. Furthermore, *O*-GlcNAcylation modulates ferroptosis by targeting critical regulators including SLC7A11, FTH, TFRC, YAP, and ZEB1, thereby affecting lipid peroxidation, iron metabolism, and redox homeostasis. Through OGT- and OGA-mediated cycling, *O*-GlcNAcylation acts as a metabolic sensor that integrates nutrient and stress signals to coordinate apoptotic, autophagic, and ferroptotic responses. “G” indicates *O*-GlcNAc modification sites.

Many studies indicate a negative correlation between *O*-GlcNAcylation and apoptosis, although some studies suggest the opposite (**Figure 2**). For example, inhibition of *O*-GlcNAcylation promotes doxorubicin (Dox)-induced apoptosis in HCC cells (54). Reduced *O*-GlcNAcylation of NOS1AP leads to more severe neuronal apoptosis (55). *O*-GlcNAcylation of CD36 suppresses cardiomyocyte apoptosis (56). Inhibition of *O*-GlcNAcylation of RIPK1 at Ser331, Ser440, and Ser669 enhances the formation of the RIPK1/FADD/Caspase-8 complex, thereby promoting sunitinib-induced RIPK1-dependent apoptosis (23). Conversely, some studies suggest that *O*-GlcNAcylation is positively correlated with apoptosis. For example, *O*-GlcNAcylation of nNOS promotes neuronal apoptosis during glutamate stimulation by enhancing the formation of the nNOS–postsynaptic density protein 95 (PSD-95) complex. Inhibiting *O-*GlcNAcylation reduces hyperglycemia-induced podocyte apoptosis through the ER-stress-*O-*GlcNAcylation axis (57). Overall, the interplay between *O-*GlcNAcylation and apoptosis is context-dependent, varying with target proteins, cell types, and disease conditions. Specifically, whether *O*-GlcNAcylation promotes or inhibits apoptosis depends on its effect on the target protein and, in turn, the role of that protein in apoptosis within a particular cell type and environmental context. For example, overexpression of CD36 inherently suppresses cardiomyocyte apoptosis, and *O*-GlcNAcylation of CD36 enhances its stability and expression, thereby exerting an anti-apoptotic effect (56).

### Autophagy

The interaction between autophagy and apoptosis is regulated by multiple signaling pathways and is highly dependent on the specific cellular or physiological context. Generally, autophagy functions as a cytoprotective process against apoptosis, whereas the activation of apoptosis-associated caspases suppresses autophagy. However, under specific conditions, autophagy or autophagy-related proteins may contribute to apoptosis or necrosis (58). Autophagy is the major process that mediates the transport of diverse intracellular components to lysosomes for degradation and recycling. When nutrients are scarce or cells are threatened by harmful factors such as bacteria, viruses, and oncogenes, autophagy is activated as a defense mechanism. This process begins with the release of mTOR inhibition, which allows the ULK complex and Class III PI3K complex to initiate phagophore formation. The ATG5-ATG12-ATG16 complex functions similarly to the ubiquitination machinery, promoting membrane elongation, whereas LC3 decorates the membrane to guide selective cargo sequestration. Autophagy involves cargo specificity through receptors such as p62 and NBR1, which mediate selective processes (59).

*O-*GlcNAc modification plays a pivotal role throughout the autophagic process, orchestrating autophagosome formation and autophagosome-lysosome fusion (**Figure 2**). This dynamic modification has been identified in numerous autophagy-related proteins, highlighting its importance in the fine-tuning of autophagic machinery (**Figure 1**) (60). For example, GRASP55 promotes autophagosome-lysosome fusion by facilitating the interaction between LC3-II and LAMP2, but its *O*-GlcNAcylation can inhibit this process and acts as a glucose sensor to modulate autophagosome maturation according to cellular metabolic status (61). Similarly, mutations at the *O-*GlcNAc sites of SNAP-29 enhance SNARE complex assembly, thereby promoting autophagosome–endosome/lysosome fusion and accelerating autophagic flux (62). Moreover, the autophagy-related kinases mTOR and AMPK exhibit a complex bidirectional crosstalk with *O*-GlcNAcylation, collectively contributing to the maintenance of cellular homeostasis (63). These findings suggest that *O*-GlcNAc not only regulates the fundamental processes of autophagy but may also dynamically modulate autophagic flux and cell fate by sensing nutritional and stress signals. Future studies could investigate how *O*-GlcNAc modifications differentially regulate autophagy under various pathological conditions and evaluate their potential as therapeutic targets.

### Ferroptosis

Ferroptosis, a distinct form of regulated cell death, was first proposed in 2012 (64). It is typically accompanied by lipid peroxidation and excessive iron accumulation. The characteristics of ferroptosis include mitochondrial shrinkage, reduced mitochondrial cristae, and rupture of the outer mitochondrial membrane (65). In ferroptosis, phospholipid membranes abundant in polyunsaturated fatty acids (PUFAs) undergo excessive peroxidation. In particular, acyl-CoA synthetase long-chain family member 4 (ACSL4) facilitates the binding of PUFAs to coenzyme A (CoA), whereas lysophosphatidylcholine acyltransferase 3 (LPCAT3) reintegrates PUFA-CoA into phospholipids, making them part of the cell membrane. Ferrous iron (Fe²^+^) subsequently generates reactive oxygen species (ROS) via the Fenton reaction, triggering PUFA peroxidation, leading to membrane damage (66). Glutathione peroxidase 4 (GPX4) serves as a crucial intracellular regulatory factor, playing a significant role in maintaining redox homeostasis. Research indicates that decreased GPX4 activity and the accumulation of lipid peroxides disrupt membrane structure and cellular homeostasis, ultimately leading to ferroptosis (67).

*O-*GlcNAcylation plays a key role in regulating cellular iron homeostasis and ferroptosis by modulating ferritinophagy and iron uptake pathways (**Figure 2**) (68). For example, the removal of *O*-GlcNAc from the ferritin heavy chain (FTH) at S179 promotes its binding to the ferritinophagy receptor NCOA4, thereby facilitating ferritinophagy and ferroptosis (69). In Addition, reduced *O*-GlcNAc levels compromise the stability of FTH1, leading to disrupted iron homeostasis and increased iron accumulation. In contrast, elevated *O*-GlcNAc levels stabilize ferritin and limit the release of labile iron, thereby effectively preventing ferroptosis (70). Recent evidence suggests that reactive ROS can stimulate OGT enzymatic activity, thereby promoting the O-GlcNAcylation of FOXK2. This modification enhances the interaction between FOXK2 and importin α, facilitating its nuclear translocation and subsequent binding to the SLC7A11 promoter region. As a result, SLC7A11 transcription is elevated, ultimately suppressing HCC ferroptosis (71). In summary, *O*-GlcNAcylation finely regulates cellular iron homeostasis and ferroptosis through multiple mechanisms, playing a critical role in maintaining metabolic balance and influencing disease progression. A deeper understanding of its regulatory mechanisms in specific diseases may provide a foundation for developing precise ferroptosis-targeted interventions.

*O*-GlcNAcylation dynamically modulates apoptosis, autophagy, and ferroptosis. In apoptosis, *O*-GlcNAc modification of key proteins such as CDK5, RIPK1, and AKT regulates mitochondrial outer membrane permeabilization (MOMP), caspase activation, and the balance between cell survival and death. In autophagy, *O*-GlcNAcylation of GRASP55, SNAP29, SIRT3, and PINK1 influences autophagosome formation, lysosomal fusion, and mitochondrial quality control under cellular stress. Furthermore, *O-*GlcNAcylation modulates ferroptosis by targeting critical regulators including SLC7A11, FTH, TFRC, YAP, and ZEB1, thereby affecting lipid peroxidation, iron metabolism, and redox homeostasis. Through OGT- and OGA-mediated cycling, *O*-GlcNAcylation acts as a metabolic sensor that integrates nutrient and stress signals to coordinate apoptotic, autophagic, and ferroptotic responses. “G” indicates *O*-GlcNAc modification sites.

### Pyroptosis

The phenomenon of pyroptosis was first observed in 1992 (72). Nearly a decade later, in 2001, this form of pro*-*inflammatory PCD was formally termed “pyroptosis,” to differentiate it from apoptosis, which is typically non-inflammatory (73). Pyroptosis is an inflammatory and lytic form of PCD, typically initiated by inflammasomes and executed by gasdermin proteins, a family of pore-forming proteins. The human gasdermin family consists of GSDMA, GSDMB, GSDMC, GSDMD, GSDME/DFNA5, and PVJK/DFNB59 (74). Gasdermin, the executioner of pyroptosis, is cleaved by activated caspases or granzymes to release its N-terminal domain (GSDMD-NT) (75), which subsequently forms pores in the cell membrane, leading to the release of IL-1β and IL-18, thereby amplifying inflammatory effects and activating immune responses (76, 77).

Pyroptosis can be categorized into two pathways: the caspase-1-dependent classical pathway and the caspase-1-independent non-classical pathway, the latter of which is mediated by human caspase-4/5 or mouse caspase-11 (78). Cell swelling, pore formation in the membrane, and the subsequent release of intracellular substances are the primary features of pyroptosis. Under normal physiological conditions, pyroptosis plays a crucial role in defending the host against microbial infections. For example, studies using macrophages induced to undergo pyroptosis without releasing IL-1β or IL-1α have shown that their supernatants upregulate gene signatures associated with cell migration, proliferation, and wound healing, thereby enhancing tissue repair *in vivo* (79). In addition, GSDMD deficiency in macrophages impairs tissue recovery and delays muscle regeneration (80). In summary, the pyroptotic secretome contains metabolites with tissue repair properties, which could be leveraged for therapeutic purposes. However, excessive pyroptosis can trigger uncontrolled and persistent inflammatory responses, which play a role in the progression of inflammatory diseases.

**Figure 3:**
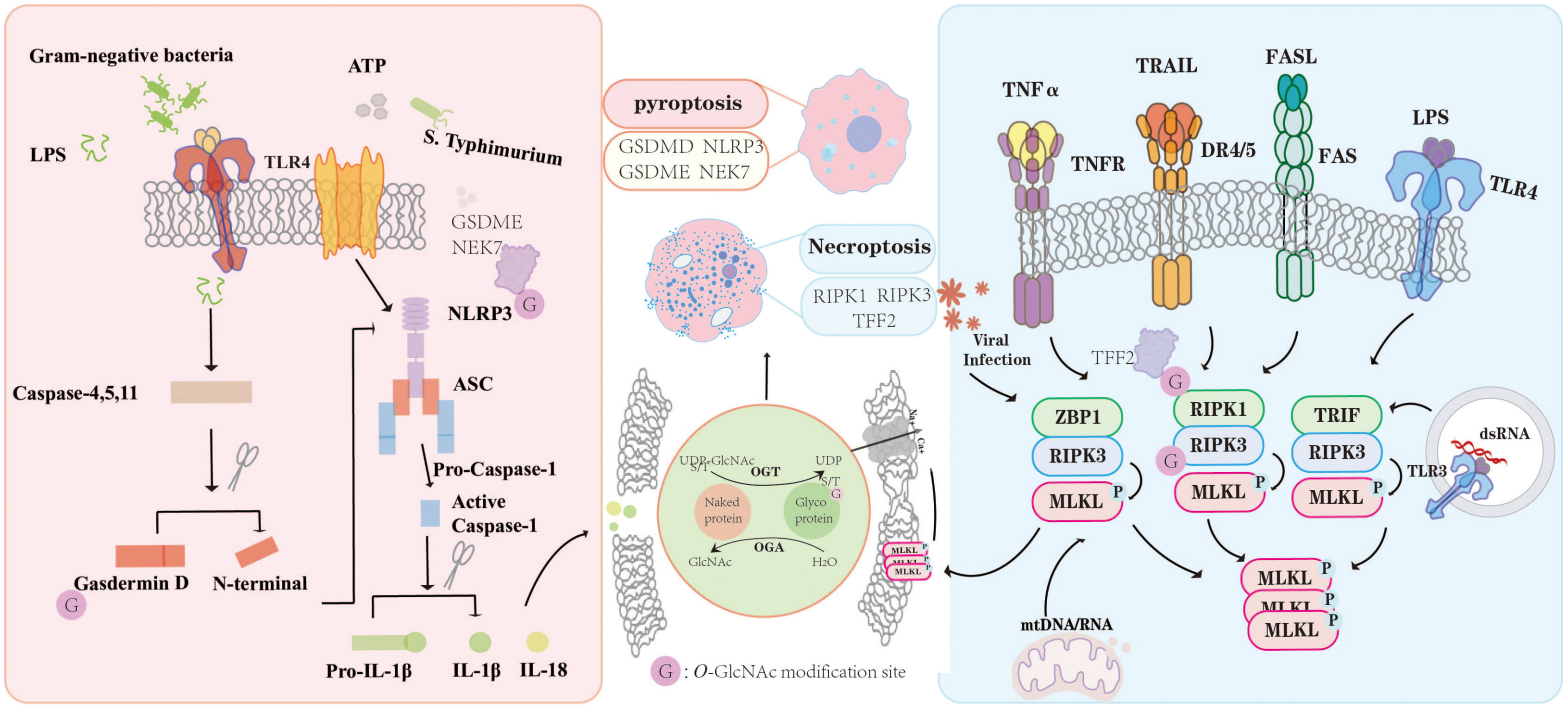
*O*-GlcNAc modification exerts multifaceted regulatory effects on PCD pathways. In the context of pyroptosis, *O*-GlcNAcylation of key signaling proteins such as NLRP3, GSDMD, GSDME, and NEK7 modulates inflammasome activation and the maturation of pro-inflammatory cytokines IL-1β and IL-18. Similarly, during necroptosis, *O*-GlcNAcylation of RIPK1, RIPK3, and TFF2 influences the assembly and phosphorylation of necrosome complexes in response to TNF-α, TRAIL, FASL, and TLR signaling. Through dynamic regulation by OGT and OGA, *O*-GlcNAcylation functions as a fine-tuning mechanism linking metabolic cues to inflammatory and cell death signaling, thereby maintaining immune homeostasis under physiological and pathological conditions. “G” indicates *O*-GlcNAc modification sites.

The role of *O-*GlcNAcylation in pyroptosis depends on target proteins (**Figure 3**). Some studies have shown that *O-*GlcNAcylation prevents pyroptosis, whereas others indicate that it promotes pyroptosis (**Table 1**). For example, *O*-GlcNAc modification of GSDMD at Ser338 has been shown to attenuate pyroptosis in HUVECs by disrupting its interaction with caspase-11 (81). Conversely, other studies have demonstrated that *O*-GlcNAcylation promotes pyroptosis and aggravates inflammation. High glucose (HG)-enhanced *O-*GlcNAcylation of GSDME at Ser339 site increased the levels of IL-1β, IL-18, TNF-α, NLRP3, GSDMD, and Caspase-1, thereby promoting pyroptosis in macrophages (82). Similarly, inhibiting the *O-*GlcNAcylation of NEK7 enhances its phosphorylation at Ser260 site, thereby blocking the interaction between NEK7 and NLRP3 and suppressing chondrocyte pyroptosis (83). These findings indicate that *O*-GlcNAcylation precisely modulates the interactions and phosphorylation status of target proteins at specific sites, thereby exerting bidirectional regulation of pyroptosis depending on the cellular context. Nevertheless, current research on the interplay between *O*-GlcNAcylation and pyroptosis remains limited, highlighting the urgent need to systematically elucidate its molecular mechanisms and functional roles in disease development and progression.

**Table 1:** The roles of *O-*GlcNAc-mediated PCD.

PCD	Model of study	Target protein	Modification site	Regulatory mechanism	Role in PCD	Functions	Refs
Apoptosis	Human bladder cancer cell line	CDK5	–	Influenced CDK5 stability	Mutation of *O-*GlcNAc site promoted apoptosis	Reduced cell proliferation in bladder cancer	(94)
	RCC cell lines and xenograft mice model	RIPK1	S331,440,669	Hindered the formation of the RIPK1/FADD/Caspase-8 complex	*O-*GlcNAc suppressed sunitinib-induced RIPK-dependent apoptosis	Induced renal cell carcinoma	(23)
	Acute kidney injury mice model	AKT	–	Activated PI3K/Akt signaling pathway	*O*-GlcNAc of AKT attenuated apoptosis	Prevented kidney injury	(43)
Autophagy	HeLa cells and NRK cells	GRASP55	–	Targeted to the autophagosome-lysosome interface through LC3-II and LAMP2	GRASP55 de-O-GlcNAcylated facilitated autophagy	GRASP55 sensed glucose levels through O-GlcNAc	(61)
	HeLa cells	SNAP-29	–	Influenced the assembly of a SNARE complex	Mutations of *O*-GlcNAc sites of SNAP-29 facilitated autophagy	Integrated nutrient status with autophagosome maturation	(62)
	SKOV3 cell and xenograft mice model	SNAP-29	–	Facilitated SNARE complex assembly	OGT knockdown enhanced cisplatin-induced autophagic flux	Overcame cisplatin resistance in ovarian cancer	(95)
	Rats model of type I diabetes	SNAP-29	–	Influenced the SNAP29-STX17-VAMP8 complex	*O*-GlcNAc-modified SNAP-29 impaired autophagy	Exacerbated myocardial injury in type I diabetic rats	(96)
	H9C2 cell	SIRT3	S190	Enhanced SIRT3 enzymatic activity	SIRT3 *O*-GlcNAc attenuated malignant autophagy	Attenuated malignant autophagy in myocardial cells during reperfusion	(97)
	SH-SY5Y cells and AD mice model	PINK1	–	Enhanced PINK1 and LC3 expression	Elevated *O*-GlcNAc significantly suppressed mitophagy	Disrupted glial homeostasis	(40)
Ferroptosis	Bel-7402 and SMMC-7721 cells	SLC7A11	–	Stabilized OGT	Inhibited *O-*GlcNAc of SLC7A11 promoted ferroptosis	Inhibited HCC	(98)
	U2OS cells	FTH	S179	Promoted FTH interaction with NCOA4	De-*O*-GlcNAc facilitated ferroptosis	–	(69)
	HepG2.2.15, Hep3B, Huh7, and LM3 cells	TFRC	S687	Enhanced TFRC stability	De-*O-*GlcNAc of TFRC facilitated ferroptosis	Inhibited HCC	(25)
	LESCs, hCECs cells and mice model	YAP	–	Interfered with K48-linked ubiquitination and stabilization of YAP	YAP *O*-GlcNAc promoted corneal epithelial cell ferroptosis	Damaged corneal epithelial cells	(99)
	Cancer cells and mice xenograft tumor models	ZEB1	S555	–	*O*-GlcNAc of ZEB1 facilitated ferroptosis	Inhibited the function of mesenchymal cancer cell	(100)
Pyroptosis	HUVECs, HEK, 293T cells, and septic mice model	GSDMD	S338	Prevented GSDMD interaction with caspase-11	*O*-GlcNAc of GSDMD mitigated LPS-induced pyroptosis	Mitigated sepsis-associated vascular endothelial injury	(81)
	HepG2 cell	NLRP3	–	Promoted the stability of NLRP3 protein	The interaction of NLRP3 and OGT induced pyroptosis	Induced lipid metabolism dysfunction	(101)
	HGFs cells	NLRP3	T542	Heightened the expression of OGT	Enhancing the *O*-GlcNAc promoted the pyroptosis	Induced the pathogenesis of periodontitis	(102)
	THP-1 monocytes	GSDME	S339	Elevated GSDME expression	*O*-GlcNAc of GSDME augmented pyroptosis	Induced periodontitis	(82)
	ATDC5 cells	NEK7	S260	Blocked the interaction between NEK7 and NLRP3	Inhibiting the *O*-GlcNAc suppressed LPS-induced chondrocyte pyroptosis	Mitigated osteoarthritis	(83)
Necroptosis	HEK293, MEF cells	RIPK1	S331	Inhibited the phosphorylation of RIPK1	Decreased *O*-GlcNAc of RIPK1 accelerated necroptosis	Alleviated erythrocyte pathology resulting from LPS-induced endotoxemia	(93)
	TNBS-Induced Colitis mice model	RIPK3	–	RIPK3 O-GlcNAc Inhibited the binding of RIPK3 and MLKL	RIPK3 *O*-GlcNAc Inhibited necroptosis	Alleviated the pathogenesis of inflammatory bowel disease	(103)
	IR mice model	RIPK3	–	Reduced the formation of RIPK3/MLKL complex	RIPK3 *O*-GlcNAc restrained necroptosis	Restrained myocardial ischemia-reperfusion injury	(104)
	HEK293T, H1299 cells, and OGT-LKO mice model	RIPK3	–	Inhibited the formation of the MLKL complex	OGT suppresses necroptotic	Restrained liver fibrosis	(92)
	LX-2 cells and OGT-LKO mice model	TFF2	–	Influenced the OGT-TFF2 axis	OGT-deficient activated hepatocyte necroptosis	Promoted the fibrogenic process	(105)
	Macrophages and septic mice model	RIPK3	T467	Prevented RIPK3-RIPK1 and RIPK3-RIPK3 interaction	*O*-GlcNAc of RIPK3 inhibited necroptosis	Suppressed inflammation in sepsis	(106)
	Primary neuronal cells	RIPK3	–	Influenced the phosphorylation of RIPK3	*O*-GlcNAc of RIPK3 Inhibited necroptosis	Inhibited necroptosis and the progression of AD	(107)

### Necroptosis

Necroptosis is a recently identified form of regulated necrosis that is driven primarily by RIPK3 and its substrate MLKL. This process critically depends on RIPK3-mediated phosphorylation of MLKL, which leads to MLKL oligomerization, translocation to the inner leaflet of the plasma membrane, and ultimately, cell lysis and death (84). Necroptosis can be triggered by a variety of upstream signals, including Toll-like receptors (85, 86), interferons, death receptors, and intracellular RNA and DNA sensors (87). Morphologically, necroptosis features disruption of the plasma membrane, swelling of intracellular organelles, and extrusion of cytoplasmic components (88). The decision between apoptosis and necroptosis is largely governed by the activity of caspase-8. When caspase-8 is inhibited, cells become susceptible to necroptosis. For instance, while the RIPK1–caspase-8 complex can mediate TNF-α-induced apoptosis, the use of caspase inhibitors diverts the cell death pathway toward necroptosis. In this context, RIPK3 is recruited to RIPK1 to form a necrosome that executes necroptosis (85). The delicate balance between Ripk1, Ripk3, and Mlkl is critical for maintaining epithelial homeostasis (89), supporting embryonic and perinatal development (90), and sustaining the survival of hematopoietic stem and progenitor cells (91).

Emerging studies suggest that *O*-GlcNAcylation modulates necroptosis by affecting the stability, phosphorylation, and interaction of RIPK1 and RIPK3 (**Figure 3**). Most studies indicate that elevated *O*-GlcNAcylation suppresses necroptosis, whereas reduced *O*-GlcNAcylation promotes its activation (**Table 1****).** For example, in hepatocyte necroptosis, OGT suppresses necroptosis and fibrosis by downregulating RIPK3 and disrupting MLKL complex assembly (92). In addition, under LPS-induced inflammatory conditions, reduced *O*-GlcNAcylation of RIPK1 at serine 331 enhances erythrocyte necroptosis by impairing phosphorylation at serine 166 and promoting RIPK1-RIPK3 complex formation (93). Although current studies have demonstrated a negative regulatory role of *O*-GlcNAcylation in necroptosis, the existing evidence remains limited. The identified *O*-GlcNAc-modified proteins are confined primarily to core components of the necroptotic pathway. Given the dynamic and broad regulatory potential of *O*-GlcNAcylation, the identification of novel target proteins may reveal diverse regulatory mechanisms in necroptosis. A classification summary of *O*-GlcNAc-mediated regulation of PCD is provided in **Table 1**.

*O*-GlcNAc modification exerts multifaceted regulatory effects on PCD pathways. In the context of pyroptosis, *O*-GlcNAcylation of key signaling proteins such as NLRP3, GSDMD, GSDME, and NEK7 modulates inflammasome activation and the maturation of pro-inflammatory cytokines IL-1β and IL-18. Similarly, during necroptosis, *O*-GlcNAcylation of RIPK1, RIPK3, and TFF2 influences the assembly and phosphorylation of necrosome complexes in response to TNF-α, TRAIL, FASL, and TLR signaling. Through dynamic regulation by OGT and OGA, *O*-GlcNAcylation functions as a fine-tuning mechanism linking metabolic cues to inflammatory and cell death signaling, thereby maintaining immune homeostasis under physiological and pathological conditions. “G” indicates O-GlcNAc modification sites.

## *O-*GlcNAc regulated programmed cell death in diseases

Aberrant *O-*GlcNAc has been implicated in various diseases, including immune-inflammatory diseases, neurodegenerative diseases, organ and tissue injury, cardiovascular diseases, cancer, and metabolic diseases. In immune-inflammatory diseases, *O-*GlcNAc modification of inflammation-related proteins influences their stability, expression, and downstream signaling pathways, thereby mediating either pro*-*inflammatory or anti-inflammatory responses. In neurodegenerative diseases, *O*-GlcNAc reduces tau accumulation and α-synuclein aggregation (108). In organ and tissue injury, cardiovascular, and metabolic diseases, *O*-GlcNAc functions as a key stress sensor that regulates cellular survival, death, energy metabolism, and repair processes (109, 110). In cancer, *O-*GlcNAc exerts either oncogenic or tumor-suppressive effects by modifying diverse target proteins and transcription factors, thereby regulating cell function and death (111). The following section further summarizes the roles and therapeutic implications of *O-*GlcNAc in diseases through its regulation of classical forms of PCD.

### *O*-GlcNAc-regulated PCD in immune-inflammatory diseases

Increasing evidence suggests that *O*-GlcNAcylation centrally regulates immune-inflammatory diseases by modulating immune cell signaling, activation, and function. When *O-*GlcNAc cycling is blocked in macrophages, it promotes macrophage polarization toward the M1 phenotype, increasing iNOS expression and subsequently elevating the gene expression of pro*-*inflammatory cytokines (112). *O*-GlcNAcylation is essential for B-cell stability and antibody responses. OGT deficiency impairs B-cell receptor (BCR) signaling, while *O*-GlcNAcylation of Lyn at Ser19 is indispensable for BCR-mediated immune activation (113). Furthermore, in T-cells, glycoproteomic analysis revealed more than 200 *O-*GlcNAc-modified proteins, many of which are related to RNA metabolism. *O-*GlcNAc plays a key role in T-cell activation by regulating signaling pathways and protein function (114).

In immune-inflammatory diseases, *O*-GlcNAcylation exerts bidirectional regulation of inflammation by modulating the release of pro-inflammatory cytokines. For example, increasing *O*-GlcNAc levels in human synovial fibroblasts or on NF-κB p65 reduces cytokine release, attenuating inflammation and cardiovascular dysfunction in sepsis (115, 116). Similarly, GlcN and PUGNAc enhance NF-κB p65 *O*-GlcNAcylation at Ser536 to alleviate TNF-α-induced inflammatory stress (117). Conversely, under HG or TNF-α stimulation, *O*-GlcNAcylation of NF-κB or TAK1 can promote pro-inflammatory cytokine production and drive M1 macrophage polarization (118–121), highlighting its context-dependent effects. MAVS, a key antiviral signaling protein, is also regulated by OGT-mediated O-GlcNAcylation, which enhances innate immunity and suppresses viral replication (122). These cells infected with bacteria or viruses may undergo apoptosis or autophagy. If apoptotic cells are not efficiently cleared, they can progress to secondary necrosis, releasing intracellular components as DAMPs, which may trigger necroptosis or pyroptosis, activate the immune system, and induce inflammation (123). In the following section, we summarize the roles of *O*-GlcNAcylation in regulating autophagy, ferroptosis, pyroptosis, and necroptosis in immune-inflammatory diseases.

#### Autophagy

In immune-inflammatory diseases, *O*-GlcNAcylation critically regulates autophagy to modulate inflammation. By modifying key autophagy-related proteins such as Beclin1, ULK1, and SNAPs, *O*-GlcNAcylation controls autophagy initiation, elongation, and autophagosome–lysosome fusion, thereby influencing immune cell function. For example, in sepsis, aberrant *O*-GlcNAcylation impairs protective autophagy and promotes the release of pro-inflammatory cytokines (113). In osteoarthritis, *O*-GlcNAcylation of GATA4 at Ser406 inhibits its degradation via p62-mediated selective autophagy, exacerbating disease progression (124). These findings highlight *O*-GlcNAc as a key regulator of the autophagy–immune axis, though its precise mechanisms require further investigation.

#### Ferroptosis

An iron-dependent form of PCD, drives inflammation in immune-inflammatory diseases such as inflammatory bowel disease and osteoarthritis by activating immune cells and promoting pro-inflammatory cytokine release (125, 126). Emerging evidence indicates that O-GlcNAcylation plays a critical role in modulating ferroptosis in OA. For example, increased O-GlcNAcylation of GPX4 suppresses ferroptosis in chondrocytes and reduces inflammation, suggesting a protective mechanism in OA (127). Conversely, OGT-mediated *O*-GlcNAcylation of ACSF2 at Ser385 promotes ferroptosis in chondrocytes, thereby aggravating OA progression (128). These findings indicate that the effects of *O*-GlcNAcylation on ferroptosis and diseases are context-dependent, determined by the specific target proteins involved.

#### Pyroptosis

Pyroptosis is a type of lytic cell death that triggers a vigorous inflammatory response and activates the inflammasome (129). *O*-GlcNAcylation plays a bidirectional role in regulating pyroptosis: in LPS-stimulated macrophages, cellular *O*-GlcNAc levels decrease, and OGT deficiency exacerbates immune activation and promotes pro-inflammatory signaling (106). However, in vascular endothelial cells, *O*-GlcNAcylation of NF-κB/p65 enhances its nuclear translocation and transcriptional activity, thereby exacerbating the release of pro-inflammatory cytokines (130). Therefore, the regulatory functions of *O*-GlcNAc in immune-inflammatory diseases cannot be straightforwardly classified as either beneficial or detrimental.

Emerging evidence highlights specific mechanisms of O-GlcNAc in pyroptosis. For instance, in sepsis models, O-GlcNAc modification of GSDMD at Ser338 disrupts its interaction with caspase-11, attenuating pyroptosis in HUVECs (81). Conversely, in periodontitis and osteoarthritis, OGT-mediated *O*-GlcNAcylation of NLRP3 or GSDME enhances pyroptosis and exacerbates inflammation (101, 102). Additionally, inhibition of NEK7 O-GlcNAcylation blocks its interaction with NLRP3, reducing chondrocyte pyroptosis and alleviating OA (83).

Therapeutically, pharmacological elevation of O-GlcNAc, for example via OGA inhibitors such as Thiamet-G (TMG), suppresses GSDMD cleavage and mitigates LPS-induced pyroptosis in sepsis (81). In contrast, under certain chronic inflammatory conditions, inhibition of *O*-GlcNAcylation, such as Ogt knockdown, reduces pyroptosis and pro-inflammatory cytokine release in chondrocytes or macrophages, representing a potential strategy for OA and periodontitis (82, 83). In summary, O-GlcNAcylation acts as a key regulator of pyroptosis by fine-tuning target protein stability and function, linking metabolic cues with inflammatory signaling, and demonstrating significant therapeutic potential in immune-inflammatory diseases. However, its precise molecular mechanisms remain to be fully elucidated.

#### Necroptosis

Accumulating evidence indicates that necroptosis plays a pivotal role in the pathogenesis of immune-inflammatory diseases, with a more pronounced pro-inflammatory effect than apoptosis (87, 131). Emerging studies reveal that *O*-GlcNAcylation serves as a critical negative regulator of necroptosis, particularly under systemic inflammatory conditions. Mechanistically, *O*-GlcNAc modification of RIPK3 has been shown to inhibit necroptotic signaling, thereby alleviating disease progression in inflammatory bowel disease models (103). Small-molecule modulators such as TMG and NButGT suppress necroptosis by increasing RIPK3 *O-*GlcNAcylation and reducing RIPK1 phosphorylation, thereby protecting erythrocytes from LPS-induced damage during endotoxemia (93). Furthermore, the traditional Chinese medicine formulation *Wumei Pill* (WMW) alleviated colitis in mice by enhancing OGT activity, thereby increasing RIPK3 *O-*GlcNAcylation, disrupting RIPK3-MLKL interactions, and suppressing necroptosis (103). Conversely, reduction of *O*-GlcNAcylation, achieved via conditional *Ogt* deletion or OSMI-1 treatment, enhances RIPK3 phosphorylation and exacerbates necroptosis in macrophages (106). Liver-specific deletion of *Ogt* further reinforced this axis, leading to excessive necroptosis and consequent development of hepatic fibrosis and portal inflammation (92). Collectively, these findings underscore the therapeutic potential of targeting *O*-GlcNAcylation to modulate necroptosis in immune-inflammatory diseases.

In addition, studies on the regulation of other forms of cell death by *O*-GlcNAcylation are limited. Some evidence suggests that cuproptosis-related metabolic imbalance can reduce *O*-GlcNAc levels, and together, these processes participate in regulating cell fate and immune homeostasis in diseases such as sepsis (132).

### *O*-GlcNAc-regulated PCD in neurodegenerative diseases

*O*-GlcNAc is highly expressed in the brain, where its dynamic regulation is essential for maintaining synaptic function and neuronal activity. Dysregulated *O*-GlcNAc metabolism has been increasingly linked to the pathogenesis of neurodegenerative disorders. In (AD), reduced *O-*GlcNAcylation promotes tau hyperphosphorylation and aggregation into neurofibrillary tangles, while OGA inhibition can restore tau *O*-GlcNAcylation, reduce abnormal phosphorylation and aggregation, improve cognitive function, and decrease amyloid-β levels (133, 134). Similarly, in Parkinson’s disease (PD), *O*-GlcNAc alleviates 6-OHDA-induced neuronal death, neuroinflammation, motor deficits, and mitochondrial dysfunction, partly by inhibiting α-synuclein aggregation at Ser87 (135–137). While these findings highlight the neuroprotective potential of *O*-GlcNAc, its precise mechanisms and long-term safety remain to be fully elucidated.

Building on these observations, recent studies have begun to explore how *O*-GlcNAc modulates multiple forms of PCD-including autophagy, pyroptosis, and necroptosis-in neurodegenerative diseases, providing new insights into its role in neuronal survival and pathology.

#### Autophagy

Emerging evidence highlights a critical role of *O*-GlcNAcylation in the regulation of autophagy in neurodegenerative diseases. In AD mouse models, activation of autophagy has been shown to suppress apoptosis, mitigate cognitive deficits via inhibition of the Fas/FasL-VDAC1 signaling axis, reduce amyloid-β (Aβ) accumulation and neurofibrillary tangle formation, and preserve hippocampal neuronal integrity (138).

*O*-GlcNAc-modifying enzymes are intimately involved in these processes, modulating mitochondrial function and key autophagy-related proteins in both AD and PD (139). Notably, in PD, excessive *O*-GlcNAcylation can activate mTOR, impair autophagic flux, and exacerbate α-synuclein accumulation (140), whereas in AD, *O*-GlcNAcylation promotes neuronal autophagy via mTOR-independent pathways, facilitating the clearance of pathological tau (141, 142). These findings highlight the critical role of *O*-GlcNAcylation in the regulation of autophagy, emphasizing the importance of elucidating its underlying mechanisms to better understand the pathogenesis of neurodegenerative diseases and to develop targeted therapeutic strategies.

#### Pyroptosis

Pyroptosis plays a key role in amplifying neuroinflammation and exacerbating central nervous system pathology, while its inhibition has been shown to promote behavioral recovery and neuronal repair (143). In AD models, *O*-GlcNAcylation of NF-κB/p65 exerts anti-inflammatory effects: restoring *O*-GlcNAcylation at Ser384 in astrocytes suppresses p65 phosphorylation and nuclear translocation, reduces Aβ plaque deposition, and ameliorates cognitive deficits by attenuating NF-κB pathway activation (144). Similarly, in PD, *O*-GlcNAcylation of NEK7 at Thr170/Thr172 inhibits its interaction with NLRP3, thereby blocking the NEK7/NLRP3 pathway and potentially mitigating disease progression (145). However, research on the regulation of pyroptosis by *O*-GlcNAcylation in neurodegenerative diseases remains limited and warrants further investigation.

#### Necroptosis

In the nervous system, necroptosis exacerbates neurodegenerative diseases such as multiple sclerosis (MS), AD, and PD (146). Emerging evidence suggests that *O*-GlcNAcylation acts as a critical regulator of necroptosis, modulating its impact on neuronal survival and neuroinflammation. In MS, the inhibition of caspase-8 and the upregulation of RIPK1, RIPK3, and MLKL drive neuroinflammation and neuronal death (147, 148), but whether O-GlcNAcylation can modulate this pathway remains unexplored. In AD, necroptosis is activated through the TNF-α/TNFR1 axis, leading to downstream RIPK1/RIPK3/MLKL cascade activation and neuronal loss (149). Importantly, *O*-GlcNAcylation of RIPK3 inhibits its phosphorylation and interaction with RIPK1, thereby suppressing necroptosis and slowing AD progression (107). In PD, necroptosis-mediated axonal degeneration contributes to disease progression, and its inhibition has neuroprotective effects (150, 151), yet the role of *O*-GlcNAcylation in this context has not been studied. Overall, these findings highlight *O*-GlcNAcylation as a potential regulator of necroptosis in neurodegenerative diseases, with clear evidence in AD and possible implications in MS and PD.

### *O*-GlcNAc-regulated PCD in organ and tissue injury

In the context of organ and tissue injury, elevated levels of *O*-GlcNAcylation regulate apoptosis, ferroptosis, and pyroptosis, thereby modulating repair and damage.

#### Apoptosis

In models of renal and intestinal injury, the small-molecule compound GlcN enhances *O*-GlcNAcylation, attenuates renal tubular damage, suppresses apoptosis, and improves overall renal function (43). Similarly, GlcN enhances flux through the HBP, elevates intestinal *O-*GlcNAc levels, and inhibits the apoptosis of intestinal epithelial cells, thereby mitigating intestinal injury, with consistent protective effects also observed with TMG treatment (152). Moreover, enhanced *O-*GlcNAcylation of AKT by GlcN prevents kidney injury by reducing apoptosis, oxidative stress, and renal dysfunction via activation of the PI3K/Akt pathway (43). Conversely, hepatocyte-specific OGT knockout sensitizes hepatocytes to oxidative and endoplasmic reticulum (ER) stress-induced apoptosis, exacerbating liver fibrosis and injury (153).

#### Ferroptosis and pyroptosis

*O*-GlcNAcylation similarly regulates ferroptosis and pyroptosis. Environmental toxins such as deoxynivalenol (DON) impair *O*-GlcNAcylation, increase ferritinophagy and ferroptosis, and cause hepatocellular damage (154). Conversely, under cigarette smoke stimulation, *O*-GlcNAcylation of YAP in corneal epithelial cells impedes its ubiquitination, thereby promoting ferroptosis, which leads to increased epithelial cell death and exacerbates corneal tissue damage (99). In spinal cord injury (SCI) models, O-GlcNAcylation modulates pyroptosis, and inhibition of OGT reduces pro-inflammatory cytokine production and pyroptotic cell death, mitigating SCI progression (155).

These outcomes highlight that appropriate restoration of *O*-GlcNAcylation levels can facilitate organ and tissue recovery; however, excessive elevation may lead to adverse effects. A clearer understanding of how *O*-GlcNAcylation dynamically modulates apoptosis, ferroptosis, and pyroptosis under specific injury or stress conditions will help guide the development of precise targeted therapeutic strategies for organ and tissue injury-related diseases.

### *O*-GlcNAc-regulated PCD in cardiovascular diseases

Cardiovascular diseases, including coronary artery disease, heart failure, hypertensive heart disease, and myocardial infarction, are prevalent and associated with high mortality, characterized by structural and functional cardiac impairments. Disruption of cardiomyocyte homeostasis and multiple forms of cell death contribute to cell loss, inflammation, and tissue remodeling, thereby exacerbating cardiac dysfunction. Elucidating the signaling pathways and regulatory networks governing these cell death processes is crucial for understanding cardiovascular diseases pathophysiology and developing novel therapeutic strategies (156). *O*-GlcNAcylation, serving as a bridge linking cellular metabolism and protein function, has emerged as a crucial regulator of cellular processes involved in maintaining cardiovascular homeostasis (157). Studies have shown that *O*-GlcNAcylation significantly influences the development and progression of cardiovascular diseases by modulating various forms of PCD.

#### Apoptosis

Evidence indicates that dysregulated *O*-GlcNAcylation contributes to the pathogenesis of cardiac hypertrophy and heart failure by modulating apoptosis and necroptosis (158). For example, OGT-mediated *O*-GlcNAcylation of CD36 at Ser195 mitigates myocardial ischemia-reperfusion (I/R) injury by promoting cardiomyocyte proliferation and suppressing apoptosis (56).

#### Autophagy

In I/R injury, elevated *O*-GlcNAcylation of SIRT3 at the S190 site enhances its enzymatic activity, leading to activation of SOD2 and attenuation of maladaptive autophagy in cardiomyocytes during reperfusion. Conversely, inhibition of SIRT3 *O*-GlcNAcylation at Ser190 exacerbates simulated I/R injury (97), highlighting its protective role in regulating autophagy.

#### Pyroptosis

Pyroptosis contributes significantly to the progression of acute myocardial infarction (AMI). Recent studies demonstrate that *O*-GlcNAcylation of GSDME at Thr94 reduces myocardial tissue injury and lowers NLRP3 and GSDME-N protein levels, thereby exerting a cardioprotective effect through suppression of cardiomyocyte pyroptosis (159).

#### Necroptosis

Necroptosis is closely associated with I/R injury, myocardial infarction, and heart failure (160). For example, leucine-rich repeat-containing G protein-coupled receptor 6 (LGR6) mitigates myocardial I/R by activating Wnt signaling and subsequently downregulating STAT2 and ZBP1, thereby inhibiting cardiomyocyte necroptosis (161). RIPK3 also contributes to myocardial injury via Ca²^+^/calmodulin-dependent protein kinase II (CaMKII)-mediated opening of the mitochondrial permeability transition pore (mPTP) (162). However, studies on the relationship between *O*-GlcNAcylation and necroptosis in cardiovascular diseases remain limited. Current evidence suggests that *O*-GlcNAcylation exerts a negative regulatory effect on necroptosis, for example, RIPK3 *O*-GlcNAcylation reduces myocardial I/R injury by preventing RIPK3-MLKL complex formation and necroptosis (104). Its specific mechanisms and regulatory patterns remain to be further investigated.

Collectively, these findings underscore that *O*-GlcNAcylation is a key modulator of multiple PCD pathways in cardiovascular diseases. Understanding how it dynamically regulates apoptosis, autophagy, pyroptosis, and necroptosis in the heart may provide new insights for developing targeted therapeutic strategies to mitigate cardiac injury and improve functional outcomes.

### *O*-GlcNAc-regulated PCD in cancer

*O-*GlcNAc plays a dual role in cancer, depending on the target protein, cell type, and microenvironment. Numerous studies have demonstrated that *O*-GlcNAcylation has emerged as a key regulator in multiple cancer types, including breast, endometrial, colorectal, lung, and pancreatic cancers (163). In breast cancer, *O-*GlcNAc modification of MITF at serine 49 promotes its interaction with importin α/β, thereby facilitating its translocation to the nucleus by suppressing senescence and promoting cell survival and proliferation (164). In endometrial cancer, *O-*GlcNAc modification promotes cell proliferation (165). In rectal cancer, *O-*GlcNAc modification of c-Myc at Ser415 enhances the stability of c-Myc and increases the expression of pyruvate dehydrogenase kinase 2 (PDK2), which reduces mitochondrial pyruvate metabolism, inhibits ROS production, and promotes the growth of xenograft tumors (166). In conclusion, this dynamic modification is intricately linked to tumor growth, invasion, drug resistance, metabolism, and immune evasion, and warrants further exploration. Multiple studies have shown that *O*-GlcNAc modification plays a critical role in cancer by regulating apoptosis, autophagy, and ferroptosis.

#### Apoptosis

In the context of cancer, most studies have demonstrated a negative regulatory relationship between *O*-GlcNAcylation and apoptosis, whereby inhibition of *O*-GlcNAcylation promotes apoptosis and suppresses cancer cell proliferation. For instance, mutation of *O-*GlcNAc site inhibited *O-*GlcNAcylation, thereby promoting apoptosis and reducing proliferation in bladder cancer cells by modulating CDK5 stability (94). OGT knockdown via sh-OGT reduces *O-*GlcNAcylation, activates endoplasmic reticulum stress, and triggers apoptosis in breast cancer cells, ultimately inhibiting tumor growth (167). Additionally, OSMI-1, by inhibiting *O*-GlcNAcylation, has also been shown to accelerate apoptosis in neuroblastoma cells (168). The chemical modification of OSMI-1 with salicylic acid-conjugated liposomes (OSMI-1-SAL) significantly improves its pro-apoptotic and anti-proliferative effects on HCC (169). Furthermore, reducing *O-*GlcNAcylation by silencing GFAT, the rate-limiting enzyme of the HBP, or using OSMI-4, promotes apoptosis in colorectal cancer cells and suppresses tumor progression (170). Nevertheless, the lack of specificity in *O*-GlcNAc-targeted modifications presents a significant obstacle for their therapeutic application.

#### Autophagy

Many studies have elucidated the critical protective role of autophagy in disease prevention and cellular homeostasis. However, the role of autophagy in tumor migration, invasion, and metastasis remains controversial. Autophagy may play dual roles in cancer, acting as either a suppressor or promoter of tumor progression (171).

*O-*GlcNAc modification plays a crucial role in the progression of cancer through the regulation of autophagy. In chemoresistant ovarian cancer tissues, *O-*GlcNAc levels are significantly lower than those in chemosensitive tissues. OGT knockdown enhances cisplatin-induced autophagic flux, promotes autolysosome formation, and facilitates SNARE complex assembly via SNAP-29. This mechanism underscores OGT as a key candidate for overcoming cisplatin resistance in ovarian cancer (95). Reducing *O-*GlcNAc levels via si-OGT or OSMI-1 promotes autophagy and enhances antitumor efficacy in multiple myeloma, in part through destabilization of CDC27 (172).However, the relationship between *O*-GlcNAcylation and autophagy is not simply negative regulation. Some studies have shown that in glioblastoma, elevated *O-*GlcNAc levels promote cellular proliferation through autophagy activation, whereas reduced *O-*GlcNAc levels suppress autophagy, induce apoptosis, and enhance the sensitivity of glioblastoma cells to the chemotherapeutic agent temozolomide (173). Similarly, OGA inhibition by PUGNAc increases ATG4B *O*-GlcNAcylation, enhancing its proteolytic activity toward LC3 and promoting autophagy in SH-SY5Y cells (174). Given the inherently complex role of autophagy in tumor biology, the regulatory effects of *O*-GlcNAcylation on tumors through autophagy are even more intricate, warranting further investigation to elucidate the underlying mechanisms.

#### Ferroptosis

The induction of ferroptosis in tumors is a promising strategy for cancer treatment. In recent years, increasing attention has been given to the regulatory role of *O*-GlcNAcylation in ferroptosis, particularly in the context of tumor initiation and progression. In general, elevated levels of *O*-GlcNAcylation are frequently associated with the suppression of ferroptosis, thereby favoring tumor cell survival, migration, and metastasis. For instance, EIF3H stabilizes OGT, increases its expression, and promotes the proliferation and invasion of HCC cells by inhibiting ferroptosis (175). Conversely, inhibiting *O-*GlcNAc promotes ferroptosis and suppresses cancer progression. Targeting USP8 reduces the *O*-GlcNAcylation of SLC7A11, thereby inducing ferroptosis and inhibiting HCC progression (176). Interestingly, under specific stress conditions, elevated *O*-GlcNAcylation may instead promote ferroptosis. For example, in mesenchymal-type pancreatic cancer cells under hyperglycemic conditions, *O*-GlcNAc modification of ZEB1 at Ser555 promotes lipid peroxidation–driven ferroptosis through the FASN-FADS2 axis (100), indicating that the role of *O*-GlcNAcylation in cancer ferroptosis depends on external stimuli as well as the function of the target protein. In summary, generally, elevated *O*-GlcNAcylation promotes cancer progression by inhibiting apoptosis, autophagy, and ferroptosis, whereas reducing its levels suppresses tumor growth by activating these PCD. Its specific effects depend on cell type, microenvironment, and the function of target proteins, highlighting the potential value of precise modulation of *O*-GlcNAcylation in cancer therapy.

### *O*-GlcNAc-regulated PCD in metabolic diseases

Metabolic diseases are a group of disorders caused by disturbances in energy or substance metabolism, mainly including diabetes, obesity, dyslipidemia, and metabolic syndrome, among which diabetes is the most representative and extensively studied type (177). Protein *O*-GlcNAcylation plays a pivotal role in maintaining normal cellular functions, and dysregulation of *O*-GlcNAcylation contributes to the pathogenesis of diseases, particularly metabolic disorders such as diabetes (178). Existing studies have shown that *O*-GlcNAcylation influences the onset and progression of metabolic disorders, particularly diabetes and its complications, by regulating apoptosis, autophagy, ferroptosis, and pyroptosis.

#### Apoptosis

Apoptosis is one of the earliest and most extensively characterized types of cell death associated with diabetes. HG have been shown to promote DRP1-mediated mitochondrial apoptosis through the upregulation of *O*-GlcNAcylation and activation of the NFATC1 signaling pathway, thereby accelerating the onset and progression of diabetic retinopathy (179). This indicates that aberrant *O*-GlcNAc signaling may amplify hyperglycemia-induced cellular stress and mitochondrial dysfunction, contributing to retinal neurovascular damage in diabetes.

#### Autophagy

In diabetes, *O-*GlcNAc modification negatively regulates autophagy. In models of β-cell dysfunction in type 2 diabetes (T2D), the loss of *O-*GlcNAc enhances autophagy in pancreatic β cells, thereby accelerating the development of hyperglycemia in mice through mTORC1 signaling (180). Moreover, in diabetic cardiomyopathy, *O*-GlcNAcylation of SNAP29 disrupts the formation of the SNAP29-STX17-VAMP8 complex that mediates autophagosome-lysosome fusion, thereby impairing autophagy-dependent degradation and exacerbating myocardial injury in type 1 diabetic rats (96). Collectively, these findings indicate that the *O*-GlcNAc modification of autophagy-related proteins critically determines the balance between adaptive and maladaptive autophagic responses during diabetes progression.

#### Ferroptosis

Recent studies have revealed that ferroptosis, an iron-dependent form of PCD characterized by lipid peroxidation, is also regulated by *O*-GlcNAcylation in metabolic diseases. Under HG and high fat conditions, aberrant *O*-GlcNAcylation promotes endothelial cell ferroptosis, leading to impaired osteogenesis-angiogenesis coupling and the development of type 2 diabetic osteoporosis (T2DOP). Conversely, interventions that restore normal *O*-GlcNAcylation levels suppress ferroptosis, thereby improving bone formation and vascular function (181). This indicates that the importance of *O*-GlcNAc homeostasis in maintaining redox balance and vascular integrity under diabetic conditions.

#### Pyroptosis

A pro-inflammatory form of PCD, has also been linked to *O*-GlcNAc signaling in metabolic diseases. Upregulation of OGT enhances the stability and *O*-GlcNAcylation of NLRP3, thereby promoting inflammasome activation, lipid metabolism dysfunction, and pyroptotic cell death, ultimately accelerating the progression of non-alcoholic fatty liver disease (NAFLD) (101). This indicates that excessive *O*-GlcNAcylation may bridge metabolic and inflammatory pathways, amplifying lipotoxicity-driven hepatocellular injury.

In summary, dysregulated *O*-GlcNAcylation orchestrates multiple forms of PCD, including apoptosis, autophagy, ferroptosis, and pyroptosis, thereby driving the onset and progression of metabolic diseases and their complications. These findings underscore the multifaceted role of *O*-GlcNAcylation as a central metabolic sensor and suggest that fine-tuning *O*-GlcNAc signaling may offer novel therapeutic opportunities for the prevention and treatment of diabetes and related metabolic disorders. Representative disease models and therapeutic approaches based on the modulation of *O*-GlcNAcylation in PCD are summarized in **Table 2**.

**Table 2:** Targeting *O*-GlcNAc-Mediated Regulation of PCD in Various Diseases.

PCD	Strategies for Modulating *O*-GlcNAc	Impact on *O*-GlcNAc	Role in PCD	Role in Diseases	Disease	Refs
Immune-inflammatory diseases						
Pyroptosis	TMG	Up	Inhibit	Alleviated LPS-induced sepsis	Sepsis	(81)
	si-OGT	Down	Inhibit	Inhibited osteoarthritis	osteoarthritis	(83)
	si-OGT	Down	Inhibit	Inhibited periodontitis	periodontitis	(82)
Necroptosis	TMG NButGT	Up	Inhibit	Protected erythrocytes during endotoxemia	endotoxemia	(93)
	WMW	Up	Inhibit	Alleviated colitis	Colitis	(103)
	OSMI-1	Down	Induce	Exacerbated septic inflammation	inflammation	(106)
Neurodegenerative diseases						
Necroptosis	OGA haploinsufficient mice	Up	Inhibit	Alleviated AD symptoms	AD	(107)
Autophagy	TMG	Up	Inhibit	Promotes α-synuclein accumulation, neuronal dysfunction	PD	(174)
Organ and tissue injury						
Apoptosis	OGT knockout (mouse model)	Down	Induce	Exacerbated liver fibrosis and injury	Liver fibrosis	(153)
Ferroptosis	DON	Down	Induce	Induced hepatic injury	Liver injury	(154)
Cardiovascular diseases						
Autophagy	TMG	Up	Inhibit	Exacerbated cardiac injury	Myocardial injury	(96)
Apoptosis	GlcN	Up	Inhibit	Alleviated IR injury	IR injury	(43, 152)
Metabolic diseases						
Apoptosis	OSMI-1	Down	Inhibit	Protected against diabetic nephropathy	Diabetes nephropathy	(57)
Autophagy	TMG	Up	Inhibit	Alleviated β-cell dysfunction in T2D	T2D	(140)
Cancer						
Apoptosis	OSMI-1-SAL	Down	Induce	Inhibited liver cancer cell proliferation	HCC	(169)
Autophagy	si-OGT OSMI-1	Down	Induce	Enhanced anti-tumor responses	multiple myeloma	(172)
Ferroptosis	USP8 EIF3H	Up	Inhibit	Promoted HCC progression	HCC	(175, 176)

## Clinical Applications and limitations of targeting *O*-GlcNAcylation

**Figure 4:**
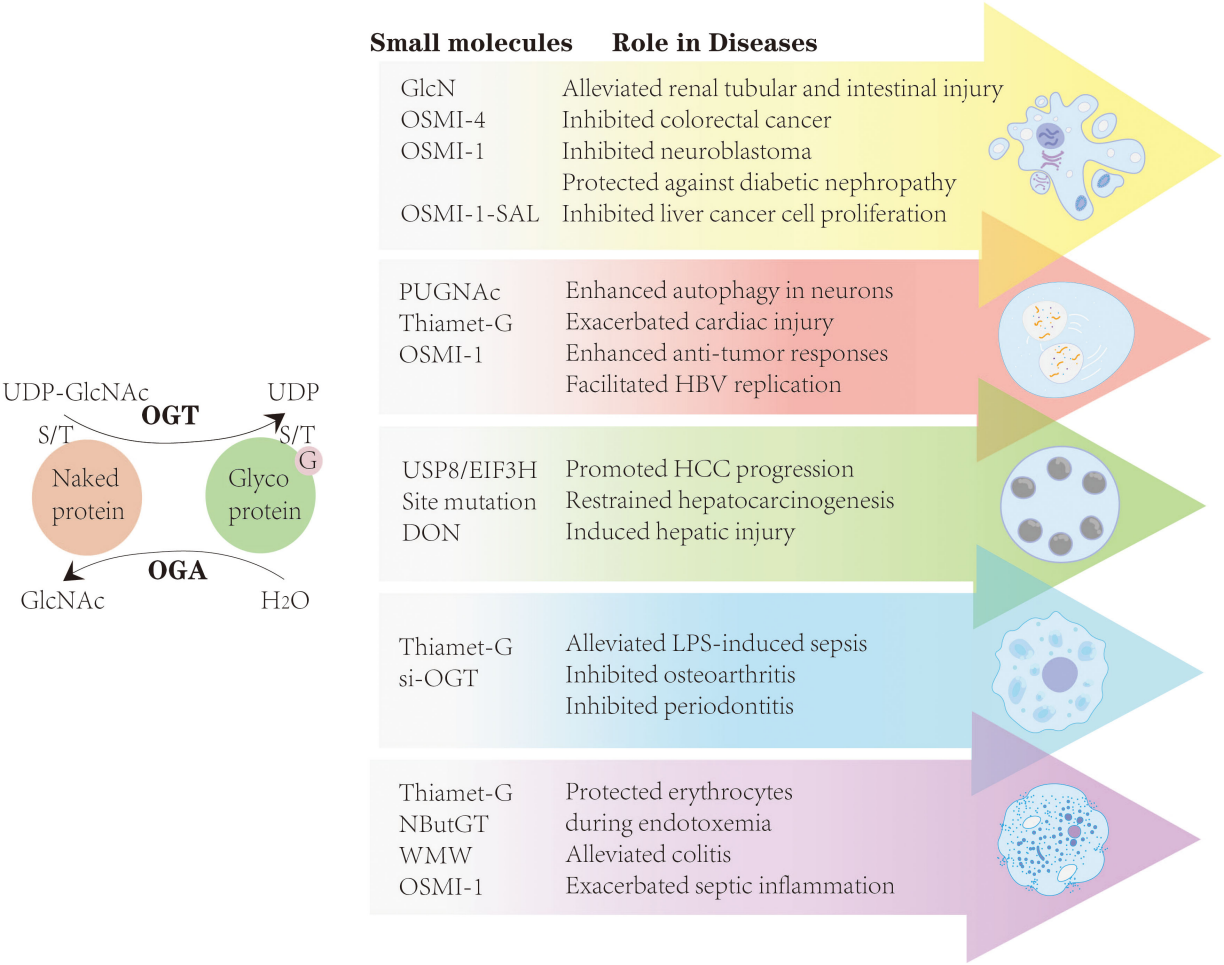
By modulating the activity of OGT and OGA, most small-molecule drugs-including TMG, OSMI-1, OSMI-4, si-OGT, and others-can influence apoptosis, autophagy, ferroptosis, pyroptosis, and necroptosis, thereby establishing a theoretical framework for targeting *O*-GlcNAcylation in diseases.

As previously discussed, *O-*GlcNAcylation is dynamically regulated by two key enzymes, OGA and OGT, and most small-molecule drugs are primarily designed to modulate the activity of these enzymes. For example, OGA inhibitors such as PUGNAc and TMG are commonly used to increase *O-*GlcNAcylation, whereas OGT activity can be suppressed via small-molecule inhibitors, including OSMI-1 and OSMI-4, or through genetic silencing tools such as shRNA-OGT and siRNA-OGT. Moreover, point mutations at specific residues, specific small-molecule compounds, and the inhibiting of GFAT, the key enzyme in the HBP, can also modulate *O-*GlcNAc levels (**Figure 4**) (182). However, OGT inhibitors, such as OSMI-1 and OSMI-4, display limited aqueous solubility and necessitate high DMSO concentrations for dissolution, hindering their applicability in mammalian models. To overcome these limitations, more water-soluble analogs, such as 5SGlcNAc, have been developed, which not only exhibit improved solubility but also efficiently penetrate cells (26).

By modulating the activity of OGT and OGA, most small-molecule drugs-including TMG, OSMI-1, OSMI-4, si-OGT, and others-can influence apoptosis, autophagy, ferroptosis, pyroptosis, and necroptosis, thereby establishing a theoretical framework for targeting *O*-GlcNAcylation in cancer, infectious diseases, and tissue repair.

Building on these mechanistic insights, recent advances in small-molecule inhibitors targeting OGA have highlighted their therapeutic potential in neurodegenerative diseases. Several OGA inhibitors, including MK-8719 (183), ASN90 (184), ASN51 (185), BIIB113 (186), and LY3372689 (187), have demonstrated favorable safety profiles and brain penetrance in early-phase clinical trials for AD, PD, progressive supranuclear palsy (PSP), and related tauopathies (188). Among them, ASN90 has progressed to Phase II trials (189), while ASN51 achieved over 95% sustained OGA enzyme occupancy in Phase I studies, indicating potent target engagement (190). Mechanistically, OGA inhibition increases tau *O*-GlcNAcylation, thereby preventing its aggregation and promoting a soluble, non-pathogenic conformation (188). Collectively, these findings suggest that fine-tuning *O*-GlcNAc signaling through OGA inhibition represents a promising therapeutic avenue for protein aggregation-driven neurodegenerative disorders, although long-term efficacy and selectivity remain critical challenges for clinical translation.

Despite these advances, there remains a lack of tools to selectively modify the *O*-GlcNAcylation of a single protein in cells without affecting other proteins. To address this, some studies have applied expressed protein ligation (EPL) to investigate *O*-GlcNAc-modified proteins *in vitro*. Chemically synthesized *O*-GlcNAc-modified peptides are ligated to bacterially expressed, unmodified protein fragments to generate full-length proteins with *O*-GlcNAc at specific sites. While this strategy enables complete site-specific modification, the resulting proteins are only applicable for *in vitro* experiments (22, 191). A recent technology employs dual-specificity RNA aptamers (DS aptamers) to achieve protein-specific *O*-GlcNAcylation in cells. DS aptamers are RNA molecules designed with one domain that binds OGT and another that binds the target protein, enabling selective *O*-GlcNAc modification of the protein. However, this approach is associated with high costs and considerable experimental complexity (27). Therefore, identifying target proteins closely associated with disease progression and performing protein-specific or site-specific *O*-GlcNAc modification, or introducing mutations at these modification sites, holds promise for precise and targeted therapeutic interventions.

## Summary and future prospects

*O-*GlcNAcylation exerts multifaceted regulatory effects on PCD by modulating target protein stability and expression, orchestrating the assembly of protein complexes, activating downstream signaling pathways, and engaging in dynamic crosstalk with other PTMs. These effects are highly context-dependent and influenced by factors such as cell type, external stimuli, specific glycosylated substrates, and the underlying pathological setting. While numerous studies have investigated the roles of *O*-GlcNAcylation in regulating individual forms of PCD or in specific disease contexts, a systematic and comprehensive synthesis across multiple PCD types and diverse diseases has been lacking. Our review fills this gap by providing the first integrative overview of how *O*-GlcNAcylation modulates apoptosis, autophagy, pyroptosis, ferroptosis, and necroptosis across a spectrum of diseases, including immune-inflammatory disorders, neurodegenerative diseases, tissue injury, cardiovascular diseases, cancer, and metabolic disorders. This provides a new perspective for developing strategies that can precisely modulate *O*-GlcNAcylation at defined sites on target proteins.

Although this review summarizes the roles and therapeutic potential of *O*-GlcNAcylation in regulating PCD across various diseases, there is currently a lack of tools that can precisely manipulate *O*-GlcNAcylation on a single protein without affecting others. Since OGT and OGA are the sole enzymes responsible for adding and removing *O*-GlcNAc, any chemical inhibition or genetic manipulation targeting them simultaneously alters the modification status of thousands of proteins. Moreover, *O*-GlcNAc and phosphorylation often occur on the same or adjacent residues in a mutually exclusive manner, and there is still no amino acid mimic analogous to phosphorylation. These factors have greatly limited the translation of basic research into clinical applications (27). Therefore, it is necessary to develop targeted therapeutics that can specifically modulate *O*-GlcNAcylation at defined sites on target proteins.

However, among the numerous *O*-GlcNAc-modified proteins involved in disease regulation, it remains to be determined which proteins engage in crosstalk and play critical roles in disease progression. Clinical trials targeting *O*-GlcNAc modification on specific proteins are limited, with most interventions focusing on global *O*-GlcNAc modulation. Therefore, future studies should explore strategies that combine targeted carriers or other materials to enable localized delivery or site-specific application of *O*-GlcNAc-targeting drugs, thereby achieving precise interventions across different diseases and multiple forms of PCD. Moreover, organoid models, which better recapitulate the *in vivo* microenvironment, offer a valuable platform to validate and refine *O*-GlcNAc-targeting strategies (192, 193). For instance, studies using 3D distal airway epithelial organoids derived from lung tissue of patients with idiopathic pulmonary fibrosis (IPF) have shown aberrant *O*-GlcNAcylation, and reduction of *O*-GlcNAc levels suppressed the expression of profibrotic genes and proteins, indicating that *O*-GlcNAc modification is a key determinant of fibrotic remodeling in organoid systems (194). Nevertheless, the application of *O*-GlcNAc modulation in organoid models remains limited, and further research is warranted.

## Glossary

*O*-GlcNAc: *O*-linked β-N-acetylglucosamine
PTM: Post-translational modification
PCD: Programmed cell death
OGT: *O*-GlcNAc transferase
OGA: *O*-GlcNAcase
GlcN: Glucosamine
GlcNAc: N-acetylglucosamine
HBP: Hexosamine biosynthetic pathway
GFAT: Glucose: Fructose-6-phosphate amidotransferase
UDP-GlcNAc: Uridine 5′-diphosphate-N-acetylglucosamine
TFRC: Transferrin receptor
mOGT: Mitochondrial isoform OGT
HK: Hexokinase
LC3: Light chain 3
PINK1: PTEN-induced kinase 1
PSD-95: Postsynaptic density protein 95
PUFAs: Polyunsaturated fatty acids
CoA: Coenzyme A
LPCAT3: Lysophosphatidylcholine acyltransferase 3
ACSL4: Acyl-CoA synthetase long-chain family member 4
GPX4: Glutathione peroxidase 4
FTH: Ferritin heavy chain
HG: High glucose
BCR: B-cell receptor
OA: Osteoarthritis
TMG: Thiamet-G
WMW: Wumei Pill
MS: Multiple sclerosis
AD: Alzheimer’s disease
PD: Parkinson’s disease
DON: Deoxynivalenol
SCI: Spinal cord injury
I/R: Ischemia-reperfusion
AMI: Acute myocardial infarction
LGR6: G protein-coupled receptor 6
CaMKII: Ca²⁺/calmodulin-dependent protein kinase II
mPTP: Mitochondrial permeability transition pore
PDK2: Pyruvate dehydrogenase kinase 2
ROS: Reactive oxygen species
OSMI-1-SAL: OSMI-1 with salicylic acid-conjugated liposomes
HCC: Hepatocellular carcinoma
T2D: Type 2 diabetes
T2DOP: Type 2 diabetic osteoporosis
NAFLD: Non-alcoholic fatty liver disease
EPL: Expressed protein ligation
DS aptamers: Dual-specificity RNA aptamers
IPF: Idiopathic pulmonary fibrosis.
